# Chemotherapy-Forward Management of Advanced Prostate Cancer: Taxane Timing, Sequencing and the Real-World Place of Immunotherapy

**DOI:** 10.3390/cancers18040648

**Published:** 2026-02-17

**Authors:** Takahide Noro, Takanobu Utsumi, Rino Ikeda, Naoki Ishitsuka, Yuta Suzuki, Shota Iijima, Yuka Sugizaki, Takatoshi Somoto, Ryo Oka, Takumi Endo, Naoto Kamiya, Hiroyoshi Suzuki

**Affiliations:** Department of Urology, Toho University Sakura Medical Center, Sakura 285-8741, Japan; takahide.noro@med.toho-u.ac.jp (T.N.); rino.ikeda@med.toho-u.ac.jp (R.I.); naoki.ishitsuka@med.toho-u.ac.jp (N.I.); yuta.suzuki@med.toho-u.ac.jp (Y.S.); shouta.iijima@med.toho-u.ac.jp (S.I.); yuuka.kizuki@med.toho-u.ac.jp (Y.S.); takatoshi.soumoto@med.toho-u.ac.jp (T.S.); ryou.oka@med.toho-u.ac.jp (R.O.); takumi.endou@med.toho-u.ac.jp (T.E.); naoto.kamiya@med.toho-u.ac.jp (N.K.); hiroyoshi.suzuki@med.toho-u.ac.jp (H.S.)

**Keywords:** cabazitaxel, docetaxel, metastatic castration-resistant prostate cancer, metastatic castration-sensitive prostate cancer, mismatch repair deficiency, microsatellite instability-high, Sipuleucel-T

## Abstract

Taxane chemotherapy remains a durable backbone in advanced prostate cancer, but its clinical value is increasingly determined by timing, sequencing, and deliverability. We synthesize pivotal randomized trials and contemporary guidance to provide a chemotherapy-forward framework spanning metastatic castration-sensitive prostate cancer (mCSPC) and metastatic castration-resistant prostate cancer (mCRPC). In mCSPC, early docetaxel added to androgen deprivation therapy—often as part of triplet intensification with an androgen receptor pathway inhibitor (ARPI)—offers the greatest absolute benefit in fit patients with high disease burden or aggressive clinical tempo. In mCRPC, docetaxel remains foundational, while cabazitaxel is preferred over ARPI switching after prior docetaxel and one ARPI, supporting mechanism-based sequencing. Practical implementation requires proactive toxicity prevention (especially neutropenia), dose and schedule individualization, and preservation of functional status to maintain eligibility for subsequent life-prolonging therapies. Immunotherapy has a limited but important niche: sipuleucel-T may benefit selected patients with low symptom burden, whereas immune checkpoint inhibitors are best reserved for biomarker-defined subsets such as microsatellite instability-high or mismatch repair-deficient tumors; tumor mutational burden should be interpreted cautiously in prostate cancer. Ongoing trials and emerging antigen-directed platforms will clarify whether chemotherapy can act as an immune-enabling partner in defined settings.

## Simple Summary

Treatment for metastatic prostate cancer has changed rapidly. Chemotherapy is no longer reserved for the end of the disease course: using the docetaxel earlier can improve survival for selected patients, and later chemotherapy can regain control when resistance to hormone-targeted drugs develops. This review provides a practical, clinic-ready framework for deciding when to use docetaxel and the second-generation taxane cabazitaxel, how to select patients based on disease burden, pace of progression, symptoms, and overall fitness, and how to deliver treatment safely with proactive supportive care. We also clarify where immunotherapy fits today. For most patients, checkpoint inhibitors have limited benefit, but they are important for specific biomarker-defined tumors, and cellular immunotherapy may help selected men with low symptom burden. Finally, we summarize ongoing trials and emerging antigen-directed immune platforms that may expand the role of immunotherapy in defined subsets.

## 1. Introduction

Systemic therapy for advanced prostate cancer has undergone a fundamental transition over the past decade, moving from predominantly sequential, hormone-centered approaches to upfront treatment intensification with multiple effective systemic agents and more individualized clinical decision-making [1,2]. This evolution builds upon earlier endocrine-therapy paradigms that long defined the therapeutic backbone of advanced disease management, whereas contemporary frameworks increasingly prioritize the selection and sequencing of systemic options according to disease burden, patient fitness, and the anticipated benefit–toxicity trade-off [3,4].

What was once a largely linear sequence—androgen deprivation therapy (ADT) followed by docetaxel and then “post-docetaxel” agents—has evolved into a continuum in which early intensification and strategic sequencing increasingly determine long-term outcomes across metastatic castration-sensitive prostate cancer (mCSPC) and metastatic castration-resistant prostate cancer (mCRPC) [5,6,7]. Contemporary overviews consistently conceptualize metastatic prostate cancer as a dynamic trajectory (mCSPC → mCRPC) in which prior exposures, resistance biology, metastatic burden, and patient fitness collectively shape the benefit–risk balance of each subsequent systemic option [5,6,7].

In parallel, the therapeutic armamentarium has expanded rapidly. Modern practice integrates androgen receptor pathway inhibitors (ARPIs), taxane chemotherapy, bone-modifying strategies, and biomarker-selected targeted therapies, while a robust developmental pipeline is advancing antigen-directed approaches (e.g., cell-surface target platforms), radioligand therapy, and other emerging immunologic strategies [5,7,8]. This breadth has improved outcomes but has also introduced practical complexity: multiple evidence-supported intensification pathways exist in mCSPC, and the optimal choice is often determined by clinical phenotype (disease volume and tempo, symptom burden, visceral involvement), patient factors (age, comorbidities, baseline functional reserve), and real-world constraints (access, monitoring capacity, and cost) [6,7,9].

Within this increasingly complex landscape, taxane-based chemotherapy remains a durable clinical backbone. Two decades of clinical experience and contemporary syntheses underscore that taxanes continue to deliver clinically meaningful disease control and survival benefit in both hormone-sensitive and castration-resistant settings, retaining particular value when rapid cytoreduction is required or when resistance to AR-targeted therapy limits subsequent options [7,9,10]. Importantly, chemotherapy’s role is no longer confined to late-line use: contemporary combination strategies in mCSPC increasingly position docetaxel earlier in the disease course for appropriately selected patients, reinforcing the principle that the timing of chemotherapy may be as consequential as the agent itself [6,7,9,10]. Concurrently, the expanding pipeline of immune and antigen-directed therapies raises a parallel question: how should chemotherapy be positioned alongside emerging immunologic strategies in a manner that is biologically rational and clinically actionable [5,8]?

Accordingly, this review focuses on chemotherapy-centered decision-making, with three aims: (i) to synthesize the evidence supporting docetaxel in mCSPC and contextualize its integration into contemporary intensification strategies; (ii) to delineate the roles of docetaxel and cabazitaxel across mCRPC with a practical emphasis on sequencing principles; and (iii) to define the real-world place of immunotherapy within a chemotherapy-forward framework, including why prostate cancer is frequently immunologically “cold,” how taxanes may interface with immune biology, and how biomarker selection may refine the use of immune-based therapies [5,6,7,8,9,10].

## 2. Where Chemotherapy Fits

Contemporary reviews underscore that the central clinical question has shifted from “Does a taxane work?” to “Which taxane, at which disease state, for which patient phenotype, and in what sequence?”, because a growing array of effective alternatives (doublets and triplets in mCSPC; multiple post-ARPI and post-taxane options in mCRPC) creates a landscape in which suboptimal timing can attenuate benefit or compromise tolerance for subsequent life-prolonging therapy [6,7]. Moreover, as taxanes are increasingly used earlier in the disease course, clinicians must anticipate that later-line efficacy and toxicity are conditioned by prior exposure, rendering longitudinal planning more consequential than single-line decisions [6,7,10].

A pragmatic framework is to position chemotherapy along three axes: (i) disease burden and tempo, (ii) symptom profile and the need for rapid cytoreduction, and (iii) patient fitness and cumulative toxicity risk [6,7,10]. In mCSPC, high-volume or biologically aggressive disease often favors early intensification strategies that include docetaxel; conversely, lower-burden disease may support ARPI-first approaches while reserving chemotherapy for later, particularly when marginal benefit may be offset by frailty or comorbidity [6,7,9]. Consistent with this phenotype-based framework, a recent meta-analysis reported reduced prostate cancer–specific mortality with docetaxel among patients with high-grade disease and low PSA levels, suggesting that this clinical phenotype may reflect aggressive tumor biology in which taxane intensification warrants consideration, while acknowledging the inherent limitations of cross-trial synthesis [11]. In mCRPC, chemotherapy may be preferred in the setting of rapid clinical progression, visceral disease, or substantial symptom burden requiring prompt disease control, and when ARPI cross-resistance or a short ARPI benefit, such as primary resistance or early progression soon after ARPI initiation, with limited clinical and/or biochemical response, suggests diminishing returns from ARPI sequencing [12,13,14]. This “phenotype-first” perspective aligns with contemporary debates in mCSPC and the practical imperative to avoid serial “trial-and-error” switching that can consume time and performance status in patients with aggressive disease [6,7,12,13,14].

### 2.1. Cost-Effectiveness and Access Considerations

Treatment selection is further shaped by real-world constraints and health-system factors. Cost-effectiveness considerations are increasingly salient in first-line mCSPC intensification, particularly as multi-agent combinations become more common and differences in drug pricing, monitoring requirements, and toxicity management influence the overall value proposition [15]. In parallel, real-world evidence demonstrates shifting practice patterns toward combination therapy in mCSPC, with increasing adoption over time and persistent variability by region, access, and institutional pathways [16,17]. These data are particularly informative because clinical trials typically enroll fitter patients with structured follow-up, whereas routine care must accommodate heterogeneous comorbidity, variable supportive-care capacity, and differences in the feasibility of delivering full-dose chemotherapy on schedule [15,16,17]. In many jurisdictions, ADT plus docetaxel is often regarded as a comparatively cost-effective intensification strategy because it entails finite-duration drug exposure and obviates prolonged daily therapy. By contrast, ARPI-containing doublets and triplets may be cost-effective in certain settings, particularly when absolute survival gains are greater, yet conclusions vary markedly according to regional pricing, reimbursement policies, monitoring intensity, and toxicity-management costs [15].

### 2.2. Practical Triggers for Early Chemotherapy

Because sequencing and prior exposure strongly condition later-line effectiveness, recent systematic syntheses and patient-journey frameworks emphasize constructing a coherent longitudinal plan rather than selecting therapies in isolation [12,13]. Practically, this entails explicitly defining (i) the intended role of chemotherapy within the individual’s “treatment arc” (early intensification versus deferred cytoreduction versus salvage), (ii) the triggers for transitioning from ARPI-based strategies to taxane therapy (symptomatic progression, visceral metastases, short ARPI benefit), and (iii) supportive-care priorities required to preserve deliverability (prophylaxis for neutropenia, neuropathy surveillance, maintenance of functional status) so that patients remain eligible for subsequent lines with proven survival benefit [12,13,14]. Guideline updates reinforce this trajectory-based approach, emphasizing alignment of therapy with disease tempo, symptoms, prior drug exposure, and patient goals rather than reliance on nominal “line of therapy” labels [14].

**Figure 1:**
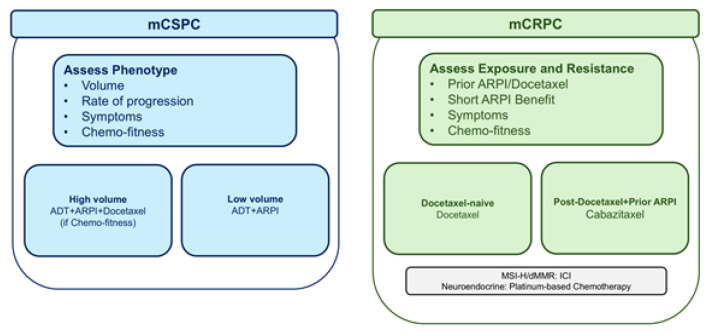
Phenotype-First Decision Framework for Taxane-based Therapy Across mCSPC and mCRPC. ADT, androgen deprivation therapy; ARPI, androgen receptor pathway inhibitor; dMMR, deficient mismatch repair; ICI, immune checkpoint inhibitor; mCSPC, metastatic castration-sensitive prostate cancer; mCRPC, metastatic castration-resistant prostate cancer; MSI-H, microsatellite instability–high.

Chemotherapy’s core roles can be summarized as follows (Figure 1):

1. Upfront intensification in mCSPC: Docetaxel added to ADT improves overall survival in appropriately selected patients and serves as a platform for triplet regimens (ADT + docetaxel + ARPI). In practice, this role is strongest when disease burden and tempo predict the greatest absolute benefit from early cytoreduction and resistance delay, and when patient fitness supports completion of planned cycles without jeopardizing subsequent therapy [18,19,20,21,22,23].
2. Foundational and salvage therapy in mCRPC: Docetaxel remains a core first taxane for many patients with mCRPC, whereas cabazitaxel is preferred over “ARPI switching” after prior docetaxel and one ARPI in common clinical scenarios. This sequencing principle has become increasingly important because many patients enter mCRPC after substantial ARPI exposure, and avoiding low-yield ARPI-to-ARPI transitions can preserve time and performance status [12,13,14].
3. Aggressive-variant contexts: Platinum-containing strategies are considered in selected aggressive-variant or neuroendocrine presentations and remain an area of active clinical development. This niche is clinically relevant because it represents a subset in which tumor biology and clinical behavior may justify intensification beyond standard AR-targeted paradigms, while prospective evidence continues to mature [12,13,14].

## 3. Docetaxel in mCSPC

### 3.1. Evidence Base for Early Docetaxel and Triplet Intensification

The central rationale for docetaxel in mCSPC is that early cytotoxic pressure can reduce macroscopic tumor burden, attenuate the emergence of resistant clones, and translate into durable survival gains when combined with ADT. This aligns with the contemporary view of mCSPC as a window in which maximal upfront disease control may reshape the subsequent trajectory toward castration resistance, rather than merely postponing progression through sequential single-agent additions [6,7,9,10]. Clinically, early docetaxel is most compelling when the anticipated absolute survival gain outweighs near-term toxicity and does not compromise the ability to deliver subsequent life-prolonging therapy as the disease evolves [6,7].

CHAARTED (E3805) provided the clearest initial evidence supporting early docetaxel, demonstrating that adding six cycles of docetaxel at ADT initiation improved overall survival versus ADT alone and establishing docetaxel as an effective intensification strategy in mCSPC [18]. Subsequent long-term analyses refined interpretation by highlighting heterogeneity of benefit according to metastatic high volume, which was defined as the presence of visceral metastases and/or four or more bone lesions of which one or more were outside the vertebra or pelvic bones, and underscoring the importance of baseline clinical phenotype in estimating absolute benefit and selecting patients most likely to tolerate and complete chemotherapy [19]. These insights were essential for translating trial-level efficacy into real-world decision-making, particularly as the mCSPC landscape expanded to include multiple competing intensification options.

The STAMPEDE platform trial confirmed a survival benefit with upfront docetaxel in a broader population initiating long-term hormone therapy, extending generalizability beyond a single-trial framework and reinforcing that docetaxel can improve outcomes when added early to standard systemic therapy [20]. In contrast, GETUG-AFU 15 provided important context for reconciling differences across trials, including the influence of trial design, sample size, subsequent therapies, and the distribution of metastatic burden on survival signals [21]. Collectively, CHAARTED, STAMPEDE, and GETUG-AFU 15 support a pragmatic conclusion reflected in contemporary reviews: early docetaxel benefits selected patients, but its value is not uniform across all mCSPC phenotypes and must be individualized according to disease burden, tempo, and fitness [6,7,9].

In the ARPI era, docetaxel has increasingly been incorporated into triplet strategies, reframing chemotherapy as a platform rather than a stand-alone intensification. PEACE-1 reported improved outcomes when abiraterone was added to ADT + docetaxel in de novo metastatic castration-sensitive disease, supporting a multi-pronged strategy that targets androgen signaling while preserving docetaxel’s cytoreductive contribution [22]. In PEACE-1, subgroup analyses suggested that the absolute benefit of triplet intensification may be more pronounced in high-volume disease than in lower-volume phenotypes, underscoring the importance of individualizing the benefit–toxicity trade-off when selecting triplet therapy [22]. ARASENS demonstrated an overall survival benefit for darolutamide added to ADT + docetaxel, providing a clear, practice-relevant evidence base for a docetaxel-anchored triplet approach in fit patients and establishing triplet therapy as a standard option for appropriately selected mCSPC populations [23]. Across contemporary syntheses and controversy discussions, these data collectively reposition docetaxel from a purely “later” therapy to a key component of early disease-course intensification, while sharpening the need for patient selection and toxicity-aware planning in routine care [6,7,9]. Key randomized evidence supporting early docetaxel and triplet intensification is summarized in Table 1.

### 3.2. Patient Selection and Real-World Implementation

A practical chemotherapy-forward approach to mCSPC begins with clinical phenotype rather than drug names, coupled with an explicit decision regarding the intended role of docetaxel: front-loaded cytoreduction and intensification versus deferral to preserve tolerability and sequencing flexibility [6,7,9,10]. Patients with higher disease burden, rapid progression tempo, symptomatic disease, or visceral metastases often have the clearest rationale for early intensification with docetaxel-containing strategies, because the absolute benefit of early maximal control is likely to be greater and the clinical consequences of undertreatment more immediate [18,19,20,21]. Conversely, in lower-burden, indolent presentations, the balance may favor ARPI-based strategies with deferred chemotherapy—particularly when comorbidity or frailty increases chemotherapy risk or when patient preferences prioritize avoidance of short-term toxicity [6,7,9].

In contemporary practice, selection between ADT + ARPI and docetaxel-anchored triplets hinges on several practical dimensions:

1. Fitness and competing toxicity profiles: baseline performance status, frailty, marrow reserve, neuropathy risk, infection vulnerability, and comorbidity (e.g., diabetes, cardiovascular disease) influence whether docetaxel can be delivered safely without jeopardizing later therapy [6,7,9,10].
2. Treatment objectives: patients with high symptom burden may prioritize rapid cytoreduction and pain control, whereas others may prioritize maintaining daily function and avoiding chemotherapy-related fatigue or neuropathy [6,7].
3. Sequencing strategy: because prior exposure shapes later-line effectiveness, clinicians must anticipate how early docetaxel may alter subsequent options in mCRPC, including the timing and feasibility of later cabazitaxel and other systemic agents [6,7,10,12,13,14].

Real-world evidence supports the feasibility and uptake of triplet strategies. Multicenter experience in Japan suggests pragmatic implementation of triplet therapy in routine care, complementing trial-level efficacy by providing insight into tolerability and deliverability outside tightly controlled trial environments [17]. In addition, U.S. longitudinal utilization analyses demonstrate increasing adoption of combination therapy in the ARPI era, highlighting evolving practice patterns and persistent variability by region and access [16]. These observations reinforce that implementation is not solely a clinical decision; it is also contingent on supportive-care resources, monitoring capacity, and institutional pathways [15,16,17].

## 4. Docetaxel in mCRPC

Docetaxel remains a cornerstone of mCRPC management, supported by robust randomized evidence demonstrating survival benefit and symptom control and establishing it as a life-prolonging therapy in advanced disease (Table 2).

TAX 327 established that docetaxel (every 3 weeks) plus prednisone improved survival and multiple clinically meaningful outcomes compared with mitoxantrone plus prednisone, cementing docetaxel as foundational chemotherapy in mCRPC [24]. SWOG 9916 likewise demonstrated superior survival with docetaxel (with estramustine) relative to mitoxantrone plus prednisone, confirming that docetaxel’s benefit extends beyond a single regimen framework and reinforcing its status as a core standard in castration-resistant disease [26]. Updated analyses of TAX 327 further delineated the durability of benefit and refined expectations regarding tolerability and outcomes in routine practice, providing more mature survival benchmarks and underscoring docetaxel’s enduring relevance despite the expanding landscape of newer systemic options [25].

However, the central clinical question is less “whether docetaxel works” than when and in whom to deploy it, given that many patients have already received ARPI and may have been exposed to docetaxel earlier in the mCSPC setting. Contemporary systematic syntheses and patient-journey frameworks emphasize that decision-making in mCRPC should be explicitly conditioned on (i) prior exposure history (which ARPI, whether docetaxel was used in mCSPC), (ii) the depth and duration of response to earlier therapy (a surrogate for resistance biology), and (iii) clinical tempo and symptom burden [12,13]. Guideline updates reinforce this trajectory-based planning, recommending that clinicians align therapy choices with disease kinetics, symptoms, and patient goals rather than relying on overly simplistic “line-of-therapy” rules, and cautioning against low-yield sequencing patterns that may erode time and performance status [14].

From a chemotherapy-forward perspective, docetaxel in mCRPC is often favored in the setting of symptomatic progression, visceral involvement, rapidly rising disease markers, or clinical features suggesting a low likelihood of meaningful benefit from ARPI switching. Importantly, docetaxel also establishes the foundation for evidence-based post-docetaxel sequencing with cabazitaxel, underscoring that docetaxel decisions should be embedded within an integrated plan that anticipates later-line needs and preserves deliverability for subsequent life-prolonging options [12,13,14].

## 5. Cabazitaxel and Sequencing

Cabazitaxel is a second-generation taxane that retains activity after docetaxel and has become central to evidence-based sequencing in mCRPC, particularly in the modern era in which many patients have received substantial ARPI exposure before (or alongside) their first taxane [12,13,14]. Conceptually, cabazitaxel addresses a common clinical inflection point: once mCRPC progresses after docetaxel and an ARPI, clinicians must decide whether to “rotate within AR-directed therapy” or to switch mechanism of action to re-establish control. Contemporary literature increasingly argues that this decision should be guided by cross-resistance patterns, clinical tempo, and the depth and duration of prior ARPI benefit, rather than nominal line-of-therapy labels [12,13].

TROPIC established cabazitaxel plus prednisone as a life-prolonging therapy after docetaxel progression compared with mitoxantrone, defining its post-docetaxel role and demonstrating that taxane switching can restore clinically meaningful benefit even in heavily pretreated disease [27]. Practically, TROPIC provided foundational evidence that cabazitaxel is not merely “another chemotherapy”, but an agent with distinct activity in docetaxel-resistant mCRPC, supporting the premise that taxane sequencing can be rational and effective when disease remains taxane-sensitive at a biological level [10,27].

Dose selection and tolerability are pivotal for real-world adoption. PROSELICA supported a reduced starting dose (20 mg/m^2^) as noninferior to 25 mg/m^2^ for overall survival in appropriate patients, enabling a more individualized balance between efficacy and toxicity—particularly relevant for older patients or those with marginal marrow reserve who might otherwise be precluded from cabazitaxel [28]. FIRSTANA compared cabazitaxel with docetaxel as first-line chemotherapy for mCRPC and clarified positioning relative to docetaxel in chemotherapy-naïve settings, reinforcing that docetaxel remains a reasonable first taxane for many patients, whereas cabazitaxel is critical as the subsequent taxane backbone [29].

Most importantly for contemporary sequencing, CARD directly addressed a highly prevalent scenario: men previously treated with docetaxel and one ARPI. CARD demonstrated that cabazitaxel outperformed switching to the alternate ARPI (abiraterone ↔ enzalutamide), providing high-level evidence that mechanism switching to cabazitaxel is preferable to ARPI “re-challenge” in this setting [30]. This trial carries substantial implications for daily practice because many patients now enter mCRPC with prior ARPI exposure from mCSPC, and the temptation to attempt another ARPI for convenience or perceived tolerability can yield low-return therapy that consumes time and performance status. Sequencing frameworks and guideline updates consistently emphasize this point: when clinical features suggest AR-indifferent progression or a short ARPI benefit, a timely transition to cabazitaxel can preserve outcomes and maintain eligibility for subsequent life-prolonging lines [12,13,14].

Beyond standard phenotypes, platinum incorporation is frequently considered for aggressive-variant or neuroendocrine-like presentations, in which AR dependence may be attenuated and rapid progression is common. A randomized phase 1–2 study of cabazitaxel plus carboplatin provides a rationale for platinum–taxane combinations in selected patients and supports biomarker-driven development in aggressive biology subsets [31]. This evidence is particularly instructive in a chemotherapy-centered review because it illustrates how clinical phenotype and inferred biology (e.g., aggressive clinical behavior, visceral disease, low PSA relative to tumor burden) can justify intensification beyond conventional AR-targeted paradigms, while acknowledging that prospective validation and optimal selection remain active questions [12,13,14,31].

Finally, network meta-analyses and contemporary reviews can inform comparative discussion of efficacy and toxicity across taxane strategies, while also highlighting heterogeneity in populations, prior exposures, and endpoints—underscoring why individualized sequencing remains essential [32]. Taken together, the current evidence supports a practical principle: cabazitaxel should be deployed proactively—rather than reserved as an exhausted last resort—when the clinical context aligns with the CARD-like phenotype, and its delivery should be optimized through dose selection and supportive care to maximize benefit across real-world patient populations (Table 3) [12,13,14,28,30].

Beyond conventional taxane sequencing, exploratory chemotherapy-based approaches have been evaluated in heavily pretreated or treatment-refractory mCRPC, including metronomic regimens (continuous low-dose scheduling) intended to enhance tolerability and potentially exploit anti-angiogenic and immunomodulatory effects; however, their contemporary role and the magnitude of benefit remain uncertain and should be regarded as investigational [33].

## 6. Practical Implementation

The effectiveness of chemotherapy in prostate cancer depends not only on agent selection but also on deliverability—preserving adequate dose intensity and timely administration while minimizing preventable complications and maintaining functional status so that patients remain eligible for subsequent life-prolonging therapies [12,13,14]. This is particularly important because multi-agent therapy is frequently introduced earlier in the disease course, and later-line options are highly contingent on performance status and marrow reserve.

### 6.1. Docetaxel Delivery Across Disease States (mCSPC and mCRPC)

For docetaxel, effective delivery requires careful attention to corticosteroid premedication, surveillance for neuropathy and fatigue, mitigation of infection risk, and proactive management of functional decline [6,7,10]. Treatment objectives differ by disease state. In mCSPC, docetaxel is often administered as a planned, time-limited course intended to maximize absolute benefit during an “intensification window”. In this setting, completing the intended cycles on schedule—when feasible—may be particularly consequential, because early disease control can shape downstream outcomes and delay transition to castration resistance [6,7,10,18,19,20,21,22,23]. Conversely, in mCRPC, docetaxel is frequently delivered with an emphasis on symptom relief, durable disease control, and preservation of performance status; here, dose reductions, schedule modifications, and early toxicity intervention may be appropriate if they sustain overall treatment continuity and maintain eligibility for subsequent-line strategies [12,13,14,24,25,26]. Randomized phase III evidence from PROSTY suggests that a biweekly docetaxel schedule (50 mg/m^2^ on days 1 and 15 of a 4-week cycle) is better tolerated and prolongs time to treatment failure compared with the standard 3-weekly 75 mg/m^2^ regimen in castration-resistant disease, providing a pragmatic alternative when toxicity risk jeopardizes treatment deliverability [34]. In the triplet setting for mHSPC, the phase III ARASAFE trial evaluated an alternative 2-weekly docetaxel schedule (50 mg/m^2^) versus the standard 3-weekly regimen in combination with darolutamide plus ADT, with the objective of improving safety while preserving efficacy; at present, these results are largely confined to conference presentations and abstract-level reporting [35].

A pragmatic approach is to define explicitly at treatment initiation: (i) the primary objective (rapid cytoreduction vs. disease stabilization), (ii) key toxicity “stop rules” (e.g., function-limiting neuropathy), and (iii) the anticipated downstream sequence (e.g., whether cabazitaxel is expected), because these decisions shape tolerance thresholds and the intensity of supportive care [12,13,14]. This approach aligns with patient-journey frameworks and guideline updates that emphasize longitudinal planning and prevention of attrition due to cumulative toxicity or delayed therapy transitions [12,13,14].

### 6.2. Cabazitaxel Safety Optimization (mCRPC): Neutropenia Prevention as a Central Lever

For cabazitaxel, neutropenia is a major, largely preventable hazard and a key determinant of real-world deliverability. Contemporary evidence supports risk stratification for severe neutropenia and structured preventive strategies that can improve safety without compromising efficacy [36]. This is clinically salient because cabazitaxel is often used in later disease stages, when marrow reserve may already be constrained by prior docetaxel, prior ARPI, bone marrow involvement, or other therapies—rendering proactive supportive care essential to maintaining planned treatment [12,13,14,28,36].

Prospective approaches to dose individualization and neutropenia prophylaxis further support personalized delivery strategies in routine care [37]. Importantly, PROSELICA provides randomized evidence supporting a reduced starting dose (20 mg/m^2^) in appropriate patients, which can be coupled with preventive supportive care to broaden eligibility for older or more vulnerable patients while preserving survival outcomes [28]. In practice, this supports a “fit-to-treatment” paradigm: select the starting dose and prophylaxis plan according to baseline risk and the clinical imperative for disease control, then adapt dynamically based on early-cycle tolerance.

### 6.3. Real-World Context: Prior Early Docetaxel and Later Taxane Performance

Because docetaxel is now frequently delivered earlier in mCSPC, subsequent taxane therapy in mCRPC has become increasingly common and clinically challenging. Real-world outcome data describing taxane performance after prior docetaxel intensification offer valuable context for counseling and expectation setting, particularly regarding the probability of benefit, the need for vigilant toxicity monitoring, and realistic treatment goals [38]. These observations complement trial evidence by capturing the complexity of routine care—heterogeneous comorbidity, variable supportive-care capacity, and differences in treatment intervals—factors also highlighted in real-world utilization analyses and economic evaluations [15,16,17].

### 6.4. Integrating Implementation with Sequencing Frameworks and Guidelines

Finally, systematic syntheses and guideline updates emphasize sequencing logic, awareness of prior exposure, and patient-centered outcomes as essential elements of mCRPC systemic therapy planning [12,13,14,32]. In practice, chemotherapy implementation should be embedded within a forward-looking plan that anticipates (i) the next effective line of therapy, (ii) toxicity risks that threaten eligibility for subsequent treatments, and (iii) patient priorities (symptom relief, function, time in hospital, caregiver burden). Within a chemotherapy-centered framework, the “success” of docetaxel or cabazitaxel is therefore measured not only by PSA declines or radiographic responses, but also by whether treatment is delivered safely and strategically to preserve the patient’s ability to benefit from the full continuum of life-prolonging therapies available [12,13,14].

## 7. Immunotherapy in Prostate Cancer: Real-World Place

### 7.1. Why Prostate Cancer Is Often an Immunologically “Cold” Tumor

Most unselected prostate cancers exhibit limited responsiveness to immune checkpoint blockade, commonly attributed to low baseline immunogenicity, relatively sparse effector T-cell infiltration, enrichment of immunosuppressive myeloid populations, and a tumor microenvironment that constrains cytotoxic function [39,40]. Contemporary syntheses emphasize that these features are not merely descriptive; they carry direct clinical implications for trial design and bedside decision-making: “all-comer” mCRPC populations are unlikely to derive meaningful benefit from ICIs without biologic enrichment (e.g., MSI-H/dMMR) or without rational combination strategies that alter the immune contexture by increasing antigen visibility, promoting T-cell trafficking, and countering myeloid-mediated suppression [39,40].

In addition, prostate cancer is frequently characterized by a dominant AR-driven lineage program, and the evolutionary pressures imposed by long-term AR-targeted therapy can generate heterogeneous resistance states in which the immune phenotype may become even less permissive (e.g., lineage plasticity, altered antigen presentation). Although the present review is chemotherapy-centered, this broader biology helps explain why immunotherapy has thus far maintained a comparatively limited footprint in prostate cancer and why future advances are expected to arise from biomarker selection and mechanism-based combinations rather than empiric checkpoint blockade [39,40]. Accordingly, the practical role of immunotherapy can be conceptualized as: (i) a biomarker-defined option in subsets with clear immune sensitivity; (ii) a research avenue in which immune-activating combinations are evaluated; and (iii) a platform for next-generation antigen-directed approaches that may circumvent key constraints of classical checkpoint blockade [39,40].

### 7.2. Immunologic Effects of Taxanes and Immunogenic Cell Death

Taxanes are not solely cytotoxic; they can influence antigen release, dendritic cell activation, and immune–tumor interactions, providing a conceptual rationale for chemo–immunotherapy combinations. The broader oncology literature on immunogenic cell death (ICD) frames cytotoxic therapy as a potential “immune primer,” capable of increasing tumor antigen availability and shaping inflammatory signaling that may enhance responsiveness to ICIs under appropriate conditions [41]. However, the same literature underscores a critical translational caveat: ICD-like features in preclinical systems do not ensure clinically meaningful synergy, because immunotherapy efficacy requires coordinated completion of multiple steps—antigen presentation, T-cell priming, trafficking, intratumoral retention, and relief of suppressive signaling—each of which may be compromised in prostate cancer [41].

Clinically, this implies that chemo–ICI combinations should be regarded not as “inevitable winners” but as testable hypotheses that require appropriate sequencing, biomarker enrichment, and careful consideration of steroid exposure, disease distribution (e.g., bone-dominant disease), and the countervailing effects of treatment-related immunosuppression (e.g., neutropenia). These considerations provide a mechanistic lens through which to interpret why large chemo–ICI trials have not yet altered standards in unselected mCRPC and why future strategies may need to pair chemotherapy with agents that more directly remodel the prostate tumor microenvironment (e.g., myeloid targeting, cytokine modulation, antigen-directed immune platforms) [39,40,41].

### 7.3. Sipuleucel-T

Sipuleucel-T remains the most established prostate cancer immunotherapy with demonstrated overall survival benefit in mCRPC [42]. Importantly, its clinical value follows a pattern distinct from many cytotoxic or targeted therapies: survival benefit may occur without marked short-term PSA declines or radiographic responses, and the effect is often best conceptualized as immune modulation of disease trajectory rather than rapid cytoreduction [42]. This distinction is particularly relevant in a chemotherapy-centered review, as it clarifies why sipuleucel-T is most often positioned in patients with preserved performance status and limited symptoms, in whom sufficient time exists for immune effects to emerge and immediate cytoreduction is not the dominant clinical priority.

Real-world registry outcomes (PROCEED) provide additional context regarding survival patterns and implementation feasibility in routine practice, including subgroup analyses that inform counseling and expectation setting [43]. Practically, an actionable positioning statement is: sipuleucel-T may be considered for selected mCRPC patients with low symptom burden and adequate functional reserve, whereas patients with rapidly progressive, symptomatic disease more often require therapies with faster onset of disease control (often chemotherapy) [42,43]. This pragmatic, “tempo-based” positioning is concordant with the phenotype-first framework applied to chemotherapy selection across the disease continuum.

### 7.4. Chemo×ICI Trials in Prostate Cancer

Multiple trials have evaluated checkpoint inhibitors alone or in combination regimens in mCRPC, and collectively they illustrate why immunotherapy has not yet emerged as a broadly applicable standard in prostate cancer (Table 4).

First, vaccine and checkpoint monotherapy approaches have underperformed in broad populations. The phase III PROSTVAC trial did not demonstrate clinical benefit in asymptomatic or minimally symptomatic mCRPC, underscoring the limitations of vaccine strategies in heterogeneous, unselected disease [44]. KEYNOTE-199 evaluated pembrolizumab monotherapy across cohorts and observed activity in a subset but overall low response rates in unselected mCRPC, reinforcing that checkpoint blockade alone is insufficient for most patients without enrichment [45]. Combination checkpoint blockade has shown signals of activity but at the cost of substantial toxicity: CheckMate 650 (nivolumab + ipilimumab) highlighted both potential responsiveness in a minority and a considerable burden of immune-related adverse events, underscoring the narrow therapeutic window in broad mCRPC populations [46]. Similarly, CA184-043 (ipilimumab after radiotherapy in post-docetaxel mCRPC) did not establish a new standard, emphasizing that immune-priming approaches require better selection and more compelling combination logic [49]. Collectively, these studies support a key clinical inference: the principal obstacle is not a complete absence of immune responsiveness, but the inability to reliably identify and enrich the responsive minority while maintaining an acceptable toxicity profile [44,45,46,49].

Against this backdrop, chemo–ICI combinations were pursued to “heat up” tumors and increase antigen availability. CheckMate 9KD explored nivolumab plus docetaxel (among other cohorts) and provided feasibility and response signals in defined settings, supporting continued development under more refined selection strategies [50]. However, KEYNOTE-921 directly tested pembrolizumab + docetaxel versus docetaxel and did not demonstrate a significant improvement in efficacy outcomes, leaving chemotherapy standards unchanged in unselected populations [47]. From a chemotherapy-forward perspective, KEYNOTE-921 is particularly informative because it indicates that simply adding PD-1 blockade to an effective cytotoxic backbone is insufficient; rather, prostate cancer likely requires enrichment (e.g., MSI-H/dMMR), immune-context modulation beyond PD-1 alone, or antigen-directed immune strategies to achieve clinically meaningful incremental benefit [39,40,41,47]. Similarly, IMbassador250 (atezolizumab plus enzalutamide versus enzalutamide) did not improve overall survival in an unselected mCRPC population, reinforcing that checkpoint-based combinations require biomarker-driven enrichment to identify the responsive minority [48].

A further step toward enrichment is illustrated by NEPTUNES, a phase II study evaluating nivolumab and ipilimumab in mCRPC selected for immunogenic features, highlighting the broader shift toward signature-based selection rather than indiscriminate enrollment [51]. This evolution parallels other tumor types: immunotherapy yields its greatest impact when patient selection and combination design are explicitly anchored to tumor immunobiology.

### 7.5. Biomarker-Defined Populations with Clearer Benefit

Unlike “all-comer” mCRPC, certain genomic contexts provide a clearer and clinically actionable rationale for immunotherapy, constituting the most defensible “real-world” immunotherapy niche in prostate cancer today.

Across solid tumors, mismatch repair deficiency (dMMR) predicts responsiveness to PD-1 blockade [52]. This biology underpinned tissue-agnostic regulatory frameworks supporting PD-1 blockade in MSI-H/dMMR tumors [53]. In prostate cancer, MSI-H/dMMR prevalence is low but clinically meaningful, and clinical series demonstrate that MSI-H/dMMR prostate cancers can respond to immune checkpoint blockade [54]. KEYNOTE-158 confirmed pembrolizumab activity in non-colorectal MSI-H/dMMR tumors and supports tumor-agnostic use when MSI-H/dMMR is present [55]. Importantly for a chemotherapy-centered review, this yields a practical directive: testing for MSI-H/dMMR (or MMR loss) should be incorporated into the systemic therapy pathway so that eligible patients are not missed when disease becomes advanced and treatment options narrow [54,55].

More recent prostate cancer–specific analyses refine expectations and help prevent overextension of pan-cancer biomarkers. Responses are enriched in MSI-H/dMMR disease, whereas TMB-high without MSI may be less predictive in prostate cohorts, underscoring that TMB should not be assumed to function uniformly across tumor types [56]. A multi-institutional case series supports practical detection strategies—including circulating tumor DNA (ctDNA) approaches—and demonstrates clinical activity of pembrolizumab in MSI-H prostate cancer identified by ctDNA, which is particularly relevant when tissue is unavailable or decalcification limits molecular testing in bone-predominant disease [57,58].

For tumor mutational burden (TMB), prospective biomarker analyses from KEYNOTE-158 linked higher TMB with improved pembrolizumab outcomes across advanced tumors [59,60], supporting accelerated tissue-agnostic approval for pembrolizumab in TMB-high solid tumors [61]. However, systematic reviews emphasize that the predictive value of TMB varies by tumor type and clinical context, and prostate cancer appears less consistently TMB-responsive than classically high-TMB malignancies [58]. Therefore, a prudent practical position is that MSI-H/dMMR represents the most reliable immunotherapy-enriching biomarker in prostate cancer, whereas TMB-high should be interpreted cautiously and ideally contextualized alongside MSI status and other clinical–molecular features [56,59,60,61].

## 8. Conclusions

Chemotherapy remains a core pillar of systemic therapy for advanced prostate cancer, yet its clinical impact is increasingly governed by timing and sequencing rather than by nominal “lines of therapy”. In mCSPC, early docetaxel—often incorporated into contemporary triplet strategies—confers meaningful benefit in appropriately selected, fit patients, particularly when disease burden and tempo favor upfront intensification. In mCRPC, docetaxel remains foundational, whereas cabazitaxel is pivotal for evidence-based sequencing after prior docetaxel and AR-targeted therapy; optimizing dose selection and supportive care is essential to preserve deliverability and maintain eligibility for subsequent lines.

Immunotherapy occupies a limited but clinically consequential niche. Sipuleucel-T is most pertinent for selected patients with low symptom burden, and checkpoint blockade is best reserved for biomarker-defined subsets in which immune sensitivity is more probable. Large chemo–checkpoint combinations have not improved standards in unselected populations, highlighting the need for more stringent enrichment and biologically rational combination strategies. Ongoing trials in aggressive-variant disease and signature-selected cohorts, together with emerging antigen-directed immune platforms, will clarify whether chemotherapy can evolve from a purely cytotoxic backbone into an immune-enabling partner within defined clinical contexts.

## Figures and Tables

**Table 1:** Pivotal randomized trials evaluating early docetaxel added to ADT ± ARPI in mCSPC.

Trial	Population	Study Arm	Primary Endpoint	Results
CHAARTED [19]	mCSPC	ADT + docetaxel (≤6 cycles) vs. ADT	OS	Median OS 57.6 vs. 47.2 months; HR 0.72. High-volume: 51.2 vs. 34.4 months; HR 0.63. Low-volume: no OS benefit (HR 1.04).
STAMPEDE (DOC comparison) [20]	High-risk locally advanced/metastatic/recurrent CSPC	SOC + docetaxel (6 cycles) vs. SOC	OS	Median OS 81 vs. 71 months; HR 0.78. In metastatic subset at entry: median OS 60 vs. 45 months; HR 0.76.
GETUG-AFU 15 [21]	mCSPC	ADT + docetaxel vs. ADT	OS	Median OS 62.1 vs. 48.6 months; HR 0.88 (not significant)
PEACE-1 [22]	mCSPC	ADT + docetaxel + abiraterone vs. ADT + docetaxel	rPFS, OS	Median rPFS 4.46 vs. 2.03 years; HR 0.50. Median OS NR vs. 4.43 years; HR 0.75.
ARASENS [23]	mCSPC	ADT + docetaxel + darolutamide vs. ADT + docetaxel	OS	Median OS NE vs. 48.9 months; HR 0.68. 4-year OS 62.7% vs. 50.4%.

ADT, androgen deprivation therapy; ARPI, androgen receptor pathway inhibitor; HR, hazard ratio; mCSPC, metastatic castration-sensitive prostate cancer; M1, metastatic disease; NE, not estimable; NR, not reached; OS, overall survival; rPFS, radiographic progression-free survival; SOC, standard of care.

**Table 2:** Landmark randomized trials establishing docetaxel as life-prolonging chemotherapy in mCRPC.

Trial	Population	STUDY Arm	Key Outcomes
TAX 327 [24,25]	mCRPC	docetaxel q3w + prednisone vs. mitoxantrone q3w + prednisone	docetaxel q3w: median OS 18.9 vs. 16.5 months; HR 0.76.
SWOG 9916 [26]	mCRPC	docetaxel + estramustine vs. mitoxantrone + prednisone	Median OS 17.5 vs. 15.6 months; HR 0.80. Longer time to progression (6.3 vs. 3.2 months).

HR, hazard ratio; mCRPC, metastatic castration-resistant prostate cancer; OS, overall survival; q3w, every 3 weeks; QoL, quality of life; SWOG, Southwest Oncology Group.

**Table 3:** Key trials defining cabazitaxel efficacy, dosing, and sequencing in mCRPC.

Trial	Setting	Study Arm	Key Outcomes
TROPIC [27]	Post-docetaxel mCRPC	Cabazitaxel 25 mg/m^2^ q3w + prednisone vs. mitoxantrone + prednisone	Median OS 15.1 vs. 12.7 months; HR 0.70. Median PFS 2.8 vs. 1.4 months.
PROSELICA [28]	Post-docetaxel mCRPC	Cabazitaxel 20 mg/m^2^ (C20) vs. 25 mg/m^2^ (C25)	Median OS 13.4 (C20) vs. 14.5 (C25); HR 1.024; noninferiority met.
FIRSTANA [29]	Chemo-naïve mCRPC	Cabazitaxel 20 or 25 mg/m^2^ vs. docetaxel 75 mg/m^2^ (all q3w + prednisone)	Median OS 24.5 (C20), 25.2 (C25), 24.3 (docetaxel); no OS superiority vs. docetaxel.
CARD [30]	Post-docetaxel + one ARPI mCRPC	Cabazitaxel 25 mg/m^2^ q3w + prednisone + G-CSF vs. abiraterone or enzalutamide switch	Imaging-based PFS 8.0 vs. 3.7 months; HR 0.54. Median OS 13.6 vs. 11.0 months; HR 0.64.

ARPI, androgen receptor pathway inhibitor; C20, cabazitaxel 20 mg/m^2^; C25, cabazitaxel 25 mg/m^2^; G-CSF, granulocyte colony-stimulating factor; HR, hazard ratio; mCRPC, metastatic castration-resistant prostate cancer; OS, overall survival; PFS, progression-free survival; q3w, every 3 weeks.

**Table 4:** Key trials of immunotherapy in prostate cancer.

Trial	Setting	Regimen	Key Outcomes
IMPACT [42]	mCRPC	Sipuleucel-T vs. placebo	Median OS 25.8 vs. 21.7 months; HR 0.78
PROSTVAC phase III [44]	Asymptomatic/minimally symptomatic mCRPC	PROSTVAC ± GM-CSF vs. placebo	No OS benefit (HR ~1.0)
KEYNOTE-199 [45]	Post-docetaxel and ≥1 endocrine targeted therapy mCRPC	Pembrolizumab monotherapy	ORR ~3–5% in measurable disease
CheckMate 650 [46]	mCRPC (pre- vs. post-chemotherapy cohorts)	Nivolumab + ipilimumab	ORR ~10–25%
KEYNOTE-921 [47]	mCRPC after ADT + one ARPI	Pembrolizumab + docetaxel vs. docetaxel	No significant OS benefit
IMbassador250 [48]	mCRPC (progressed on abiraterone)	Atezolizumab + enzalutamide vs. enzalutamide	No OS benefit; HR 1.12

ADT, androgen deprivation therapy; ARPI, androgen receptor pathway inhibitor; GM-CSF, granulocyte-macrophage colony-stimulating factor; HR, hazard ratio; mCRPC, metastatic castration-resistant prostate cancer; ORR, objective response rate; OS, overall survival.

## Data Availability

No new data were created or analyzed in this study. Data sharing is not applicable to this article.

## Abbreviations

The following abbreviations are used in this manuscript:

ADT	androgen deprivation therapy
ARPI	androgen receptor pathway inhibitor
ctDNA	circulating tumor DNA
dMMR	mismatch repair deficiency
ICD	immunogenic cell death
mCRPC	metastatic castration-resistant prostate cancer
mCSPC	metastatic castration-sensitive prostate cancer
TMB	tumor mutational burden

## References

[1] Chakrabarti D., Albertsen P., Adkins A., Kishan A., Murthy V., Parker C., Pathmanathan A., Reid A., Sartor O., Van As N. (2025). The contemporary management of prostate cancer. CA Cancer J. Clin..

[2] Cilento M.A., Butler L.M., Emmett L., Sweeney C.J. (2025). Personalized intensification of treatment for hormone-sensitive prostate cancer. Nat. Rev. Clin. Oncol..

[3] Suzuki H., Kamiya N., Imamoto T., Kawamura K., Yano M., Takano M., Utsumi T., Naya Y., Ichikawa T. (2008). Current topics and perspectives relating to hormone therapy for prostate cancer. Int. J. Clin. Oncol..

[4] Noro T., Utsumi T., Ikeda R., Ishitsuka N., Suzuki Y., Iijima S., Sugizaki Y., Somoto T., Oka R., Endo T. (2025). Risk-adapted durations of hormone therapy combined with radiotherapy for prostate cancer. Acad. Oncol..

[5] Posdzich P., Darr C., Hilser T., Wahl M., Herrmann K., Hadaschik B., Grünwald V. (2023). Metastatic Prostate Cancer—A Review of Current Treatment Options and Promising New Approaches. Cancers.

[6] Hussain M., Fizazi K., Shore N.D., Heidegger I., Smith M.R., Tombal B., Saad F. (2024). Metastatic Hormone-Sensitive Prostate Cancer and Combination Treatment Outcomes. JAMA Oncol..

[7] Tsung I., Yentz S.E., Reichert Z.R. (2025). Controversies in metastatic hormone-sensitive prostate cancer. Cancer.

[8] Zhang J., Chadha J.S. (2024). Developmental Therapeutics in Metastatic Prostate Cancer: New Targets and New Strategies. Cancers.

[9] Kwon W.-A., Song Y.S., Lee M.-K. (2024). Strategic Advances in Combination Therapy for Metastatic Castration-Sensitive Prostate Cancer: Current Insights and Future Perspectives. Cancers.

[10] Carceles-Cordon M., Rodriguez-Bravo V., Petrylak D.P., Domingo-Domenech J. (2025). 20 years of taxane therapy in prostate cancer —the past, present and future. Nat. Rev. Urol..

[11] Mahal B.A., Kwak L., Xie W., Eastham J.A., James N.D., Sandler H.M., Feng F.Y., Brihoum M., Fizazi K., Sweeney C. (2023). Mortality Risk for Docetaxel-Treated, High-Grade Prostate Cancer With Low PSA Levels. JAMA Netw. Open.

[12] Francini E., Agarwal N., Castro E., Cheng H.H., Chi K.N., Clarke N., Mateo J., Rathkopf D., Saad F., Tombal B. (2024). Intensification Approaches and Treatment Sequencing in Metastatic Castration-resistant Prostate Cancer: A Systematic Review. Eur. Urol..

[13] McManus H.D., Dorff T., Morgans A.K., Sartor O., Shore N., Armstrong A.J. (2024). Navigating therapeutic sequencing in the metastatic castration-resistant prostate cancer patient journey. Prostate Cancer Prostatic Dis..

[14] Garje R., Bin Riaz I., Naqvi S.A.A., Rumble R.B., Taplin M.-E., Kungel T.M., Herchenhorn D., Zhang T., Beckermann K.E., Vapiwala N. (2025). Systemic Therapy in Patients With Metastatic Castration-Resistant Prostate Cancer: ASCO Guideline Update. J. Clin. Oncol..

[15] Ávila C., González-Montero J., Rojas C.I., Madan R.A., Burotto M. (2025). Current landscape in first-line treatment of metastatic hormone sensitive prostate cancer: A cost-effectiveness focused review. Oncology.

[16] Raval A.D., Lunacsek O., Korn M.J., Littleton N., Constantinovici N., George D.J. (2025). Real-World Evidence of Combination Therapy Use in Metastatic Hormone-Sensitive Prostate Cancer in the United States From 2017 to 2023. JCO Oncol. Pr..

[17] Urabe F., Imai Y., Goto Y., Tashiro K., Hashimoto M., Yoshihara K., Yamamoto S., Hara S., Miyajima K., Fukuokaya W. (2024). Real-world evidence of triplet therapy efficacy in patients with metastatic castration-sensitive prostate cancer: A Japanese multicenter study. Ultrasound Med. Biol..

[18] Sweeney C.J., Chen Y.-H., Carducci M., Liu G., Jarrard D.F., Eisenberger M., Wong Y.-N., Hahn N., Kohli M., Cooney M.M. (2015). Chemohormonal Therapy in Metastatic Hormone-Sensitive Prostate Cancer. N. Engl. J. Med..

[19] Kyriakopoulos C.E., Chen Y.-H., Carducci M.A., Liu G., Jarrard D.F., Hahn N.M., Shevrin D.H., Dreicer R., Hussain M., Eisenberger M. (2018). Chemohormonal Therapy in Metastatic Hormone-Sensitive Prostate Cancer: Long-Term Survival Analysis of the Randomized Phase III E3805 CHAARTED Trial. J. Clin. Oncol..

[20] James N.D., Sydes M.R., Clarke N.W., Mason M.D., Dearnaley D.P., Spears M.R., Ritchie A.W.S., Parker C.C., Russell J.M., Attard G. (2016). Addition of docetaxel, zoledronic acid, or both to first-line long-term hormone therapy in prostate cancer (STAMPEDE): Survival results from an adaptive, multiarm, multistage, platform randomised controlled trial. Lancet.

[21] Gravis G., Boher J.-M., Joly F., Soulié M., Albiges L., Priou F., Latorzeff I., Delva R., Krakowski I., Laguerre B. (2016). Androgen Deprivation Therapy (ADT) Plus Docetaxel Versus ADT Alone in Metastatic Non castrate Prostate Cancer: Impact of Metastatic Burden and Long-term Survival Analysis of the Randomized Phase 3 GETUG-AFU15 Trial. Eur. Urol..

[22] Fizazi K., Foulon S., Carles J., Roubaud G., McDermott R., Fléchon A., Tombal B., Supiot S., Berthold D., Ronchin P. (2022). Abiraterone plus prednisone added to androgen deprivation therapy and docetaxel in de novo metastatic castration-sensitive prostate cancer (PEACE-1): A multicentre, open-label, randomised, phase 3 study with a 2 × 2 factorial design. Lancet.

[23] Smith M.R., Hussain M., Saad F., Fizazi K., Sternberg C.N., Crawford E.D., Kopyltsov E., Park C.H., Alekseev B., Montesa-Pino Á. (2022). Darolutamide and Survival in Metastatic, Hormone-Sensitive Prostate Cancer. N. Engl. J. Med..

[24] Tannock I.F., De Wit R., Berry W.R., Horti J., Pluzanska A., Chi K.N., Oudard S., Théodore C., James N.D., Turesson I. (2004). Docetaxel plus Prednisone or Mitoxantrone plus Prednisone for Advanced Prostate Cancer. N. Engl. J. Med..

[25] Berthold D.R., Pond G.R., Soban F., de Wit R., Eisenberger M., Tannock I.F. (2008). Docetaxel Plus Prednisone or Mitoxantrone Plus Prednisone for Advanced Prostate Cancer: Updated Survival in the TAX 327 Study. J. Clin. Oncol..

[26] Petrylak D.P., Tangen C.M., Hussain M.H., Lara P.N.J., Jones J.A., Taplin M.E., Burch P.A., Berry D., Moinpour C., Kohli M. (2004). Docetaxel and Estramustine Compared with Mitoxantrone and Prednisone for Advanced Refractory Prostate Cancer. N. Engl. J. Med..

[27] de Bono J.S., Oudard S., Ozguroglu M., Hansen S., Machiels J.-P., Kocak I., Gravis G., Bodrogi I., Mackenzie M.J., Shen L. (2010). Prednisone plus cabazitaxel or mitoxantrone for metastatic castration-resistant prostate cancer progressing after docetaxel treatment: A randomised open-label trial. Lancet.

[28] Eisenberger M., Hardy-Bessard A.-C., Kim C.S., Géczi L., Ford D., Mourey L., Carles J., Parente P., Font A., Kacso G. (2017). Phase III Study Comparing a Reduced Dose of Cabazitaxel (20 mg/m^2^) and the Currently Approved Dose (25 mg/m^2^) in Postdocetaxel Patients With Metastatic Castration-Resistant Prostate Cancer—PROSELICA. J. Clin. Oncol..

[29] Oudard S., Fizazi K., Sengeløv L., Daugaard G., Saad F., Hansen S., Hjälm-Eriksson M., Jassem J., Thiery-Vuillemin A., Caffo O. (2017). Cabazitaxel Versus Docetaxel As First-Line Therapy for Patients With Metastatic Castration-Resistant Prostate Cancer: A Randomized Phase III Trial—FIRSTANA. J. Clin. Oncol..

[30] de Wit R., de Bono J., Sternberg C.N., Fizazi K., Tombal B., Wülfing C., Kramer G., Eymard J.-C., Bamias A., Carles J. (2019). Cabazitaxel versus Abiraterone or Enzalutamide in Metastatic Prostate Cancer. N. Engl. J. Med..

[31] Corn P.G., Heath E.I., Zurita A., Ramesh N., Xiao L., Sei E., Li-Ning-Tapia E., Tu S.-M., Subudhi S.K., Wang J. (2019). Cabazitaxel plus carboplatin for the treatment of men with metastatic castration-resistant prostate cancers: A randomised, open-label, phase 1–2 trial. Lancet Oncol..

[32] Shao C., Wan Q., Guo J., Chen Z. (2025). The efficacy and safety of cabazitaxel in the treatment of metastatic castration-resistant prostate cancer: A systematic review and network meta-analysis based on randomized controlled trials. Front. Pharmacol..

[33] Wysocki P.J., Lubas M.T., Wysocka M.L. (2022). Metronomic Chemotherapy in Prostate Cancer. J. Clin. Med..

[34] Kellokumpu-Lehtinen P.-L., Harmenberg U., Joensuu T., McDermott R., Hervonen P., Ginman C., Luukkaa M., Nyandoto P., Hemminki A., Nilsson S. (2013). 2-weekly versus 3-weekly docetaxel to treat castration-resistant advanced prostate cancer: A randomised, phase 3 trial. Lancet Oncol..

[35] Grimm M.-O., Von Amsberg G., Heers H., Degener S., Roghmann F., Casuscelli J., Rausch S., Augustin M., Häberle L., Leucht K. (2025). LBA92 3-weekly docetaxel 75 mg/m^2^ vs. 2-weekly docetaxel 50 mg/m^2^ in combination with darolutamide + ADT in patients with mHSPC: Results from the randomised phase III ARASAFE trial. Ann. Oncol..

[36] Agema B.C., Buck S.A., Viskil M., Isebia K.T., de Neijs M.J., Sassen S.D., Koch B.C., Joerger M., de Wit R., Koolen S.L. (2023). Early Identification of Patients at Risk of Cabazitaxel-induced Severe Neutropenia. Eur. Urol. Oncol..

[37] Omlin A., Cathomas R., von Amsberg G., Reuter C., Feyerabend S., Loidl W., Boegemann M., Lorch A., Heidenreich A., Tsaur I. (2023). Randomized Phase II Cabazitaxel Dose Individualization and Neutropenia Prevention Trial in Patients with Metastatic Castration-Resistant Prostate Cancer. Clin. Cancer Res..

[38] Robin G., Basappa N.S., North S., Ghosh S., Kolinsky M. (2024). Outcomes of First Subsequent Taxane Therapy in Patients with Metastatic Castration-Resistant Prostate Cancer Who Previously Received Docetaxel Intensification for Metastatic Castration-Sensitive Prostate Cancer. Curr. Oncol..

[39] Novysedlak R., Guney M., Al Khouri M., Bartolini R., Foley L.K., Benesova I., Ozaniak A., Novak V., Vesely S., Pacas P. (2024). The Immune Microenvironment in Prostate Cancer: A Comprehensive Review. Oncology.

[40] Reimers M.A., Slane K.E., Pachynski R.K. (2019). Immunotherapy in Metastatic Castration-Resistant Prostate Cancer: Past and Future Strategies for Optimization. Curr. Urol. Rep..

[41] Catanzaro E., Beltrán-Visiedo M., Galluzzi L., Krysko D.V. (2024). Immunogenicity of cell death and cancer immunotherapy with immune checkpoint inhibitors. Cell. Mol. Immunol..

[42] Kantoff P.W., Higano C.S., Shore N.D., Berger E.R., Small E.J., Penson D.F., Redfern C.H., Ferrari A.C., Dreicer R., Sims R.B. (2010). Sipuleucel-T Immunotherapy for Castration-Resistant Prostate Cancer. N. Engl. J. Med..

[43] Sartor O., Armstrong A.J., Ahaghotu C., McLeod D.G., Cooperberg M.R., Penson D.F., Kantoff P.W., Vogelzang N.J., Hussain A., Pieczonka C.M. (2020). Survival of African-American and Caucasian men after sipuleucel-T immunotherapy: Outcomes from the PROCEED registry. Prostate Cancer Prostatic Dis..

[44] Gulley J.L., Borre M., Vogelzang N.J., Ng S., Agarwal N., Parker C.C., Pook D.W., Rathenborg P., Flaig T.W., Carles J. (2019). Phase III Trial of PROSTVAC in Asymptomatic or Minimally Symptomatic Metastatic Castration-Resistant Prostate Cancer. J. Clin. Oncol..

[45] Antonarakis E.S., Piulats J.M., Gross-Goupil M., Goh J., Ojamaa K., Hoimes C.J., Vaishampayan U., Berger R., Sezer A., Alanko T. (2020). Pembrolizumab for Treatment-Refractory Metastatic Castration-Resistant Prostate Cancer: Multicohort, Open-Label Phase II KEYNOTE-199 Study. J. Clin. Oncol..

[46] Sharma P., Pachynski R.K., Narayan V., Fléchon A., Gravis G., Galsky M.D., Mahammedi H., Patnaik A., Subudhi S.K., Ciprotti M. (2020). Nivolumab Plus Ipilimumab for Metastatic Castration-Resistant Prostate Cancer: Preliminary Analysis of Patients in the CheckMate 650 Trial. Cancer Cell.

[47] Petrylak D.P., Ratta R., Matsubara N., Korbenfeld E., Gafanov R., Mourey L., Todenhöfer T., Gurney H., Kramer G., Bergman A.M. (2025). Pembrolizumab Plus Docetaxel Versus Docetaxel for Previously Treated Metastatic Castration-Resistant Prostate Cancer: The Randomized, Double-Blind, Phase III KEYNOTE-921 Trial. J. Clin. Oncol..

[48] Powles T., Yuen K.C., Gillessen S., Kadel E.E., Rathkopf D., Matsubara N., Drake C.G., Fizazi K., Piulats J.M., Wysocki P.J. (2022). Atezolizumab with enzalutamide versus enzalutamide alone in metastatic castration-resistant prostate cancer: A randomized phase 3 trial. Nat. Med..

[49] Kwon E.D., Drake C.G., Scher H.I., Fizazi K., Bossi A., Van den Eertwegh A.J.M., Krainer M., Houede N., Santos R., Mahammedi H. (2014). Ipilimumab versus placebo after radiotherapy in patients with metastatic castration-resistant prostate cancer that had progressed after docetaxel chemotherapy (CA184-043): A multicentre, randomised, double-blind, phase 3 trial. Lancet Oncol..

[50] Fizazi K., Mella P.G., Castellano D., Minatta J.N., Kalebasty A.R., Shaffer D., Limón J.C.V., López H.M.S., Armstrong A.J., Horvath L. (2022). Nivolumab plus docetaxel in patients with chemotherapy-naïve metastatic castration-resistant prostate cancer: Results from the phase II CheckMate 9KD trial. Eur. J. Cancer.

[51] Leone G., Wong Y.N.S., Jones R.J., Sankey P., Josephs D.H., Crabb S.J., Harris L., Zarkar A., Protheroe A., Vasudev N. (2025). Nivolumab and Ipilimumab for Metastatic Castration-Resistant Prostate Cancer With an Immunogenic Signature: The Multicenter, Two-Cohort, Phase II NEPTUNES Study. J. Clin. Oncol..

[52] Le D.T., Durham J.N., Smith K.N., Wang H., Bartlett B.R., Aulakh L.K., Lu S., Kemberling H., Wilt C., Luber B.S. (2017). Mismatch repair deficiency predicts response of solid tumors to PD-1 blockade. Science.

[53] Marcus L., Lemery S.J., Keegan P., Pazdur R. (2019). FDA Approval Summary: Pembrolizumab for the Treatment of Microsatellite Instability-High Solid Tumors. Clin. Cancer Res..

[54] Abida W., Cheng M.L., Armenia J., Middha S., Autio K.A., Vargas H.A., Rathkopf D., Morris M.J., Danila D.C., Slovin S.F. (2019). Analysis of the Prevalence of Microsatellite Instability in Prostate Cancer and Response to Immune Checkpoint Blockade. JAMA Oncol..

[55] Marabelle A., Le D.T., Ascierto P.A., Di Giacomo A.M., De Jesus-Acosta A., Delord J.-P., Geva R., Gottfried M., Penel N., Hansen A.R. (2020). Efficacy of Pembrolizumab in Patients With Noncolorectal High Microsatellite Instability/Mismatch Repair–Deficient Cancer: Results From the Phase II KEYNOTE-158 Study. J. Clin. Oncol..

[56] Lenis A.T., Ravichandran V., Brown S., Alam S.M., Katims A., Truong H., Reisz P.A., Vasselman S., Nweji B., Autio K.A. (2024). Microsatellite Instability, Tumor Mutational Burden, and Response to Immune Checkpoint Blockade in Patients with Prostate Cancer. Clin. Cancer Res..

[57] Somoto T., Utsumi T., Ikeda R., Ishitsuka N., Noro T., Suzuki Y., Iijima S., Sugizaki Y., Oka R., Endo T. (2025). Precision Care for Hereditary Urologic Cancers: Genetic Testing, Counseling, Surveillance, and Therapeutic Implications. Curr. Oncol..

[58] Barata P., Agarwal N., Nussenzveig R., Gerendash B., Jaeger E., Hatton W., Ledet E., Lewis B., Layton J., Babiker H. (2020). Clinical activity of pembrolizumab in metastatic prostate cancer with microsatellite instability high (MSI-H) detected by circulating tumor DNA. J. Immunother. Cancer.

[59] Marabelle A., Fakih M., Lopez J., Shah M., Shapira-Frommer R., Nakagawa K., Chung H.C., Kindler H.L., Lopez-Martin J.A., Miller W.H. (2020). Association of tumour mutational burden with outcomes in patients with advanced solid tumours treated with pembrolizumab: Prospective biomarker analysis of the multicohort, open-label, phase 2 KEYNOTE-158 study. Lancet Oncol..

[60] Zgura A., Chipuc S., Bacalbasa N., Haineala B., Rodica A., Sebastian V. (2025). Evaluating Tumour Mutational Burden as a Key Biomarker in Personalized Cancer Immunotherapy: A Pan-Cancer Systematic Review. Cancers.

[61] Marcus L., Fashoyin-Aje L.A., Donoghue M., Yuan M., Rodriguez L., Gallagher P.S., Philip R., Ghosh S., Theoret M.R., Beaver J.A. (2021). FDA Approval Summary: Pembrolizumab for the Treatment of Tumor Mutational Burden–High Solid Tumors. Clin. Cancer Res..

